# Addressing nutritional gaps with multivitamin and mineral supplements

**DOI:** 10.1186/1475-2891-13-72

**Published:** 2014-07-15

**Authors:** Elizabeth Ward

**Affiliations:** 1 24 Oak Street, 01867 Reading, MA, USA

**Keywords:** Multivitamin and mineral supplements, Vitamins, Minerals, Dietary supplements, Benefits, Risks

## Abstract

A balanced and varied diet is the best source of essential vitamins and minerals; however, nutrient deficiencies occur, including in populations with bountiful food supplies and the means to procure nutrient-rich foods. For example, the typical American diet bears little resemblance to what experts recommend for fruit, vegetables, and whole grains, which serve as important sources of an array of vitamins and minerals. With time, deficiencies in one or more micronutrients may lead to serious health issues. A common reason people take multivitamin and mineral (MVM) supplements is to maintain or improve health, but research examining the effectiveness of MVMs in the prevention of certain chronic conditions is ongoing. In addition to the utility of MVMs for filling in relatively small but critical nutritional gaps, which may help prevent conditions such as anemia, neural tube defects, and osteoporosis, some evidence supports possible benefits of MVM supplementation with regard to cancer prevention (particularly in men) and prevention or delay of cataract, as well as some aspects of cognitive performance. Unlike some single-vitamin supplements, MVM supplements are generally well tolerated and do not appear to increase the risk of mortality, cerebrovascular disease, or heart failure. The potential benefits of MVM supplements likely outweigh any risk in the general population and may be particularly beneficial for older people.

## Introduction

Evidence suggests that eating patterns that include relatively high intakes of fruit, vegetables, nuts, and whole grains are linked to a significantly lower risk of heart disease, cancer, and stroke [1,2], conditions that rank among the top 4 leading causes of death in adults living in the United States [3]. Plant foods, lean protein foods, and low-fat dairy products are all important sources of micronutrients that help ensure health and prevent disease [4].

The US Food and Nutrition Board of the Institute of Medicine (IOM) has defined Dietary Reference Intakes (DRI) for 29 vitamins and minerals (Table 1) [5]. Expert groups including the Academy of Nutrition and Dietetics (AND) recommend obtaining essential micronutrients by eating a balanced and varied diet [6]. However, persistent or periodic nutritional gaps are common in the general population [7], and people who don’t consume adequate amounts of certain foods may have nutrient shortfalls. In addition, there are times throughout the life cycle when the body requires more nutrients than the typical diet may provide, such as iron during pregnancy and vitamin B_12_ after age 50 years [4]. Over the course of a lifetime, deficiencies in one or more nutrients may contribute to serious health issues [8].

**Table 1:** **Dietary reference intakes (DRIs): recommended dietary allowances or adequate intakes for vitamins and elements **[5]

**Micronutrient**	**RDAs (bold) or AI (plain type) for adults**
**VITAMINS**	
**Vitamin A**	**700–900 μg/d**
**Vitamin C**	**75–90 mg/d**
**Vitamin D**	**15–20 μg/d**
**Vitamin E**	**15 mg/d**
Vitamin K	90–120 μg/d
**Thiamin**	**1.1–1.2 mg/d**
**Riboflavin**	**1.1–1.3 mg/d**
**Niacin**	**14–16 mg/d**
**Vitamin B**_**6**_	**1.3–1.7 mg/d**
**Folate**	**400 μg/d**
**Vitamin B**_**12**_	**2.4 μg/d**
Pantothenic acid	5 mg/d
Biotin	30 μg/d
Choline	425–550 mg/d
**ELEMENTS**	
**Calcium**	**1,000–1,200 mg/d**
Chromium	20–35 μg/d
**Copper**	**900 μg/d**
Fluoride	3-4 mg/d
**Iodine**	**150 μg/d**
**Iron**	**8–18 mg/d**
**Magnesium**	**310–420 mg/d**
Manganese	1.8–2.3 mg/d
**Molybdenum**	**45 μg/d**
**Phosphorus**	**700 mg/d**
**Selenium**	**55 μg/d**
**Zinc**	**8–11 mg/d**
Potassium	4.7 g/d
Sodium	1.2–1.5 g/d
Chloride	1.8–2.3 g/d

Dietary supplement use is common among consumers; in the National Health and Nutrition Examination Survey (NHANES), approximately half of all non-institutionalized civilian persons living in the United States were taking dietary supplements, most commonly multivitamin and mineral (MVM) supplements, for a variety of reasons [9,10]. Thirty-three percent to 39% of the total US population takes multivitamins [9,11]. In spite of their popularity, there is no standardized or regulatory definition for MVM supplements. A range of definitions have been used to describe MVMs, and the term "MVM" may refer to products of widely varied compositions and characteristics [12]. The US National Institutes of Health has defined MVMs as supplements that consist of 3 or more micronutrients at doses less than the Tolerable Upper Level (UL) determined by the Food and Nutrition Board of the IOM and are free of herbs, hormones, or drugs [13]. Other definitions suggest that MVM content should not be limited to only the B vitamins [14,15]. Using these definitions, products with widely varying compositions, ranging from those that contain a small number of vitamins or minerals to those containing many more vitamins and minerals, and in varying doses, are all classified as MVM supplements.

This article reviews the potential health benefits and risks of MVM supplements in several key areas, including cancer, cardiovascular disease, age-related eye disease, and cognition. Although the focus of the article is on MVM supplements containing 10 or more vitamins and minerals, there have been relatively few studies, such as the Physicians’ Health Study II (PHS II) [16,17], of such formulations. Most studies have investigated individual vitamins, or MVMs with only a few constituents; such studies are reviewed here as appropriate.

### Ensuring nutritional adequacy

Micronutrients are required for nearly all metabolic and developmental bodily processes. The 2010 Dietary Guidelines for Americans (DGA), issued by the US Department of Agriculture and US Department of Health and Human Services, recommend that “*nutrient needs should be met primarily through consuming foods*”, but also indicate that “*in certain cases*, *fortified foods and dietary supplements may be useful in providing one or more nutrients that otherwise might be consumed in less than recommended amounts*” [4]. The DGA cites 4 nutrients of concern in adults and children living in the United States: potassium, fiber, calcium, and vitamin D. These nutrients are routinely underconsumed in the general population. The DGA also recognizes folic acid, iron, and vitamin B_12_ as nutrients of concern in certain populations; folic acid and iron in women of childbearing age, and vitamin B_12_ in men and women over the age of 50 years [4].

The term “hidden hunger” has been used to describe nutritional deficiencies that occur when people consume adequate calories but inadequate micronutrients. “Hidden hunger” is largely due to eating patterns dominated by energy-dense, but nutrient-poor, foods that are often relatively inexpensive [18]. Large nationally representative surveys indicate that the intake of certain nutrients in the US falls below the IOM’s Estimated Average Requirement (EAR)—the average daily nutrient intake level estimated to meet the requirements of half the healthy individuals in a particular life stage and gender group [19]. For example, in an analysis of data from NHANES for 2003 through 2006, the population had total usual intakes from all food sources (excluding supplements) below the EAR for vitamins A, C, D, and E (45%, 37%, 93%, 91%, respectively), calcium (49%), and magnesium (55%) [20].

Some people are at risk of micronutrient deficiencies due to excessive losses (e.g., through hemodialysis), abnormal metabolism (e.g., genetic polymorphisms, alcoholism, conditions that impair fat absorption), and/or inadequate synthesis (e.g., insufficient sunlight exposure to allow vitamin D synthesis) [21]. Inadequate micronutrient intake, sometimes even at borderline levels of deficiency, has been linked to stunted growth and neurocognitive deficits [18], as well as increased risks of various symptoms and conditions [8,21-23]. Most nutrients act in all tissues, and all tissues need all nutrients; therefore, inadequate intakes may adversely affect every body system, but with more pronounced effects in some than others [24].

Although MVM supplement formulations vary widely in number of nutrients; dose of each; and the type, form, or source of vitamins and minerals, as a whole they are often instrumental in filling nutritional gaps [7], including in populations where the food supply is relatively bountiful and balanced [7,25-28]. In most cases, MVM supplements lack potassium and provide far less than the recommended daily intake for calcium, but many MVMs provide adequate folic acid, iron, vitamin B_12_, and vitamin D that can help prevent anemia, neural tube defects, and bone disease [29]. For example, in general, MVMs provide 100% of the daily value (a simplified reference value established by the US Food and Drug Administration to facilitate label comparisons of the nutrient content of food products [30]) for vitamin D [4], which is useful for people who consume inadequate amounts of vitamin D-rich foods, such as salmon, fortified milk, and orange juice [4].

MVM supplements are what the name implies and should not be regarded as substitutes for a balanced diet and other beneficial lifestyle habits [8]. It is worth noting that nutritional inadequacies are less common among those who take MVM supplements, not only because of the nutrients that MVMs provide, but also possibly because MVM supplement users may eat more nutrient-rich foods and may have healthier lifestyles overall [31]. Data from NHANES found that men, women, and children who used MVM supplements had higher dietary intake of key micronutrients than nonusers [7,32]. In another national US study, regular use of supplements resulted in an estimated ≥75% decrease in the proportion of older persons with inadequate micronutrient intakes [25].

### Prevention of chronic diseases

As defined by the Dietary Supplement Health and Education Act of 1994 [33], dietary supplements are intended to supplement the diet—not prevent or treat disease. Yet, according to a recent national survey of US residents, just 22% of dietary supplement users said they take supplements “to supplement the diet” [10]. Among the most common reasons people cited for using dietary supplements were to “improve overall health” and to “maintain health” [10]. The potential benefits of MVM supplements in preventing certain chronic conditions are not well established, but insight into the issue continues to emerge. When evaluating results across different studies of supplementation with different MVM formulations, it is important to note that MVM products are not homogeneous [29]. The different types and amounts of nutrients in the products available, as well as differences in the duration of supplement use make it challenging to compare across studies.

### Cancer

The studies evaluating the effects of individual or small combinations of vitamin and mineral supplements on cancer risk are inconsistent [34-40], and some trials and meta-analyses have suggested an *increased* incidence of cancer associated with certain *individual* vitamin supplements. Although β-carotene and lutein are phytonutrients rather than vitamins, their long-term use, as well as long-term retinol use, has been associated with an increased risk of lung cancer, particularly in those at high risk [41-43]. The randomized Selenium and Vitamin E Cancer Prevention Trial (SELECT) reported that high-dosage vitamin E supplementation was associated with a 17% increased risk of prostate cancer in healthy men after 7 years (*P* = .008) [44]. A meta-analysis of 6 randomized, controlled trials (RCTs) of folic acid supplementation reported a possible increased risk of any cancer [45], although another meta-analysis found folic acid supplementation to be associated with 40% to 50% reductions in risks of esophageal, gastric, and pancreatic cancers [46]. Investigations of the effects of folate on colorectal cancer risk have had conflicting results, with protective effects shown for dietary folate and effects ranging from modestly protective to potentially harmful associated with supplementation with folic acid (the synthetic form of folate) [47-52].

While findings on the effect of individual or small combination supplements on cancer risk have showed mixed results, some evidence from RCTs suggests a benefit. The French Supplémentation en Vitamines et Minéraux Antioxydants (SU.VI.MAX) RCT evaluated a supplement containing ascorbic acid 120 mg, vitamin E 30 mg, β-carotene 6 mg, selenium 100 µg, and zinc 20 mg. This supplement was associated with a 31% reduction in overall cancer incidence (*P* = .008) and a 37% reduction in overall mortality (*P* = .02) in men (ages 45–60 years), but not in women (ages 35–60 years), after a median intake of about 7.5 years [53].

Two randomized, double-blind clinical trials evaluating the effects of MVM supplements on cancer risk were conducted in rural Linxian County, China, which has a relatively high rate of esophageal cancer and related mortality, as well as a high rate of borderline deficiencies in micronutrient intake [54]. One of the Linxian trials, conducted in relatively healthy persons, found that supplementation with a combination of β-carotene 15 mg/d, vitamin E 30 mg/d, and selenium 50 μg/d for 5 years was associated with a trend toward a 7% lower risk of cancer and significant reductions in mortality (9% overall; 13% cancer specific) [54]. Long-term follow-up of this study indicated that the benefits of taking MVMs persisted for up to 10 years after active supplementation had ceased [55]. The second Linxian study compared Centrum® multivitamins (Pfizer Consumer Healthcare, Madison, NJ) (2 pills daily containing 26 vitamins and minerals) plus 15 mg/d β-carotene versus placebo for 6 years in 3,318 patients with esophageal dysplasia and no history of previous cancer [56]. MVM supplements did not reduce or increase the incidence of any cancer, including esophageal/gastric cancers, although there were nonsignificant trends favoring supplementation with regard to overall and cancer-specific mortality.

**Figure 1:**
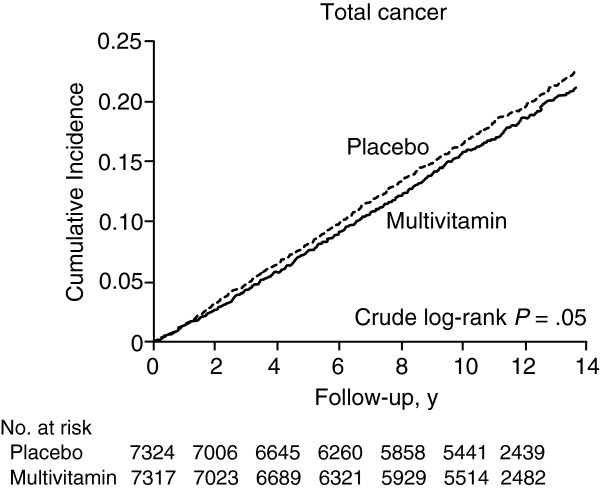
**Physicians' Health Study II cumulative incidence of total cancer with MVM supplements vs placebo **[16]**.**

The recent PHS II was a large (N = 14,641), long-term (median 11.2 years follow-up) RCT of the MVM Centrum® Silver (Pfizer Consumer Healthcare) once daily in male physicians in the United States [16]. MVM supplementation was associated with a statistically significant 8% reduction in incidence of total cancer (*P* = .04; Figure 1). In addition, men with a history of cancer derived the most benefit from MVM supplementation, with a 27% lower incidence of new cancer versus placebo in this subgroup (*P* = .02). There was no effect on the risk of any specific individual cancer types due to a lack of statistical power, and there was a nonsignificant trend toward lower cancer-specific mortality (*P* = .07). Because the study was conducted with well-nourished male physicians, it is not known whether the results apply to other types of people, including women and those more likely to have dietary insufficiencies.

Data from some meta-analyses and large nonrandomized trials of MVM supplement users also suggest a possible protective effect against cancer. A meta-analysis of 13 prospective European and North American cohort studies reported a decrease in risk of colon cancer among MVM supplement users compared with nonusers (relative risk [RR]: 0.88; 95% confidence interval [CI]: 0.81–0.96) [57]. MVM supplement use for 15 years was associated with a 75% reduction in colon cancer risk in the prospective Nurses’ Health Study (NHS) based on questionnaires completed by 88,756 female nurses in the United States [51]. In another report from the NHS, MVM supplementation had no overall effect on risk of breast cancer [58]. This study did, however, report an increase in risk of non-Hodgkin’s lymphoma (6–9 multivitamins/week: RR: 1.39; 95% CI: 1.05–1.83; ≥10 multivitamins/week: RR: 3.36; 95% CI: 1.93–5.86; *P* for trend = .0008), and risk was higher among women who used MVM supplements for ≥10 years [59]. No such association was found among men in the Health Professionals Follow-up Study [59].

Overall, there is little evidence that MVM supplements increase cancer risk. Analysis of data from a 1997 dietary questionnaire administered to the Swedish Mammography Cohort reported a 19% higher risk of breast cancer among MVM supplement users versus nonusers [60]. However, a subsequent meta-analysis of 5 cohort studies (including the Swedish Mammography Cohort study) and 3 case–control studies found that MVM supplement use had no impact—positive or negative—on the risk of breast cancer (cohort studies: RR: 0.99; 95% CI: 0.60–1.60; case–control studies: odds ratio [OR]: 1.00; 95% CI: 0.51–1.97) [61]. Furthermore, the Women’s Health Initiative, a large observational study, reported that MVM supplement use was not associated with an increase in risk of breast, colorectal, endometrial, ovarian, kidney, bladder, stomach, or lung cancer in postmenopausal women [62]. A meta-analysis of 4 RCTs, 8 cohort studies, and 2 case–control studies found no association between use of MVM supplements and prostate cancer (OR: 1.11; 95% CI: 0.95–1.29) [63]. As already discussed, an increased risk of lung cancer and prostate cancer has been observed in some trials employing levels of certain micronutrients much higher than the RDA [41-45]. It is possible that the increase in cancer seen in these studies is due to the fact that high levels of individual vitamins may be harmful and should be used with caution. This theory is illustrated in SELECT, which found no association between the combination of vitamin E and selenium and prostate cancer (hazard ratio [HR]: 1.05; 99% CI: 0.89–1.22, *P* = .46), even though vitamin E was associated with an increased risk [44].

Although many of these earlier trials, including cohort studies and trials of MVM supplements with fewer than 10 ingredients, have yielded mixed results in terms of their impact on cancer incidence and mortality, PHS II confirms the results from a range of trials that suggest a benefit of dietary supplementation in cancer prevention, at least in a population of well-nourished males in the United States. PHS II was likely able to measure a prevention benefit in cancer for a number of reasons. Firstly, a multivitamin composed of most of the essential vitamins and minerals was used, thus addressing all potential nutritional inadequacies and providing appropriate balance to the different components of the multivitamin [17], since it is possible that there are potential risks associated with supplementation of single vitamins and/or minerals without combination with other complementary vitamins/minerals [44]. Secondly, PHS II employed a large population in a prospective, randomized, placebo-controlled trial and was powered to detect differences in cancer incidence. Finally, subjects in PHS II were followed for a relatively long period of time (mean of 11.2 years). As demonstrated in the NHS, long-term use of continuous multivitamins for 10 to 15 years may be required for the benefits to be realized [51].

### Cardiovascular disease

There is minimal evidence that supplementation with individual micronutrients reduces cardiovascular disease (CVD) risk. Research into potential cardiovascular benefits of dietary supplements has focused particularly on B vitamins because of their established correlation with levels of homocysteine, a marker for CVD risk, including ischemic stroke [64]. A meta-analysis of 19 RCTs of B vitamins (including folic acid, vitamin B_6_, vitamin B_12_, and B-complex vitamins) found no effect of supplementation on rates of CVD, coronary heart disease, myocardial infarction (MI), cardiovascular death, or all-cause mortality despite significant reductions in homocysteine levels; however, risk of stroke was reduced by 12% (RR: 0.88; 95% CI: 0.82–0.95) [64]. A second meta-analysis of 26 RCTs of folic acid supplementation resulted in a 7% reduction in risk of stroke (*P* = .05) [65]. Supplementation with vitamin E, β-carotene, and vitamin C, a group of vitamins sometimes referred to as antioxidant vitamins, has had similarly neutral results with regard to cardiovascular outcomes. A meta-analysis of 15 RCTs found that supplementation with vitamin E, β-carotene, and vitamin C had no effect on rates of major cardiovascular events, MI, stroke, overall or cardiovascular mortality, revascularization, coronary heart disease, angina, or congestive heart failure [66].

The double-blind, randomized, placebo-controlled Heart Outcomes Prevention Evaluation (HOPE) and 7-year open-label HOPE-The Ongoing Outcomes extension found significant increases in risk of heart failure (*P* = .03) and related hospitalization (*P* = .045) associated with vitamin E supplementation (400 IU/d) in high-risk people ≥55 years of age [67]. A meta-analysis of 9 RCTs further found vitamin E supplementation to be associated with a significant 22% increase in risk of hemorrhagic stroke (*P* = .045), but a 10% decrease in risk of ischemic stroke (*P* = .02) [68].

A number of studies have investigated the effect of MVM supplements on CVD risk. The randomized PHS II study described above also evaluated the effects of MVM supplementation on major cardiovascular events, including nonfatal MI, nonfatal stroke, cardiovascular mortality, total MI, total stroke, and congestive heart failure and found no significant reduction in CVD risk [17]. Similar results were seen in the first PHS, in which MVM supplements were not associated with any increased or decreased risk of death from coronary heart disease (*P* = .88) or CVD mortality (*P* = .46) in healthy male physicians [69].

MVM supplements were associated with cardiovascular benefits in the Stockholm Heart Epidemiology Program (SHEEP), a large Swedish population-based case–control study [70]. SHEEP compared adults aged 45 to 70 years who had no previous history of MI with those who had experienced a first MI and survived >28 days. Regular use of dietary supplements, 80% of which were MVM supplements, was associated with a significant 22% reduction in risk of MI in men and a significant 33% reduction in risk of MI in women compared with nonuse after controlling for consumption of fruit, vegetables, and fiber. However, it is difficult to draw conclusions as to the role of MVMs in this study since their use was self-reported and the composition of MVMs and other supplements used may have been somewhat variable.

The benefits of taking MVMs for women were also seen in a separate population-based Swedish cohort study (N = 38,984 women) in which use of MVM supplements alone was associated with a 27% (95% CI: 7%–43%) lower risk of MI compared with nonuse in women ages 49 to 83 years with no history of CVD. MVM supplement use for 5 years or longer was associated with a further 41% (95% CI: 44%–80%) reduction in risk of MI. However, MVM supplements had a neutral effect on risk of MI in women with a history of CVD [71].

The Vitamin and Lifestyle Study, a population-based prospective study, evaluated mortality in a cohort of 77,673 subjects from western Washington State who used either MVM supplements (defined as containing 10 or more vitamins and/or minerals) or individual vitamin C and E supplements [72]. There was no association between MVM supplement use and risk of total mortality at any duration or frequency of use, nor were MVM supplement users at a statistically significant higher risk for death from CVD, cancer, or other causes. Use of MVM supplements for 6 to 7 days per week for 10 years was associated with a decrease in mortality due to CVD among persons with no history of CVD (HR: 0.78; 95% CI: 0.62–0.98).

Results of the studies reviewed here indicate that there is little evidence that MVM supplements have a negative impact on cardiovascular risk and that they may have a modest benefit in certain groups of people.

### Age-related eye disease

Studies of 1 or 2 vitamin supplements have not proven benefit in preventing or slowing eye disease [73,74]. For example, in a Swedish population-based cohort study of men ages 45 to 79 years, use of high-dose vitamin C or E supplements was associated with an *increased* incidence of age-related cataract. However, use of multivitamins only or multiple supplements in addition to vitamin C or E was not associated with cataract risk [73].

In the randomized Age-Related Eye Disease Study (AREDS), over a median of 6.3 years, high doses of 3 vitamins with antioxidant properties (500 mg/d vitamin C, 400 IU/d vitamin E, and 15 mg/d β-carotene) with zinc (80 mg zinc oxide) significantly reduced the risk of progression to advanced age-related macular degeneration (AMD) by 28% (*P* = .007) [75] and reduced the risk of any lens opacity by 16% and of nuclear cataract by 25% (both *P* < .01) [76]. In the AREDS2 RCT, the addition of the carotenoid alcohol phytonutrients lutein (10 mg) and zeaxanthin (2 mg), and omega-3 fatty acids, docosahexaenoic acid (350 mg) and eicosapentaenoic acid (650 mg) or all four to the original AREDS formulation did not slow progression to advanced AMD or loss of visual acuity in people aged 50 to 85 years at high risk of progression to advanced AMD [77]. However, among the two-thirds of participants in the original AREDS trial who elected to take a formulation of Centrum® without lutein, MVM supplementation resulted in a significant reduction in development or progression of cataract after a median follow-up of 6.3 years (OR: 0.88; 95% CI: 0.79–0.98) [76]. Furthermore, in the 1.5-year, multicenter Age Related Macular Degeneration Study Group study, OcuGuard® (Twinlab Corporation, American Fork, UT), an MVM supplement containing 14 antioxidants and minerals, stabilized multiple measures of visual function (Logarithm of the Minimum Angle of Resolution [LogMAR] activity, near M print acuity, and 6 cycle/degree contrast sensitivity in left eyes) in patients with AMD [78].

The Italian-American Clinical Trial of Nutritional Supplements and Age-Related Cataract (CTNS) was a single-center RCT of MVM supplements (Centrum®) in persons aged 55 to 75 years who had early cataracts (n = 710) or no cataracts (n = 310) [79]. After a mean follow-up of 9 years, those who received MVM supplementation were 18% less likely to experience a lens event, defined as development or progression of any cataract (*P* = .03). Analysis of specific types of opacities indicated that MVM supplements were associated with a 34% lower risk of nuclear opacity (*P* = .004), a neutral effect on cortical opacity (*P* = .07), and a doubling in the risk of the less common posterior subcapsular cataract opacity (*P* < .001).

Similar results were seen in the PHS II trial, which also evaluated the effects of MVM supplements on eye disease in healthy male physicians [80]. This study showed that daily supplementation with MVM supplements significantly reduced the risk of total cataract by 9% compared to placebo (*P* = .04) but had no effect on the incidence of visually significant AMD [HR: 1.19; 95% CI: 0.94–1.50; *P* = .15]). As with the Italian-American CTNS, MVM supplements significantly reduced the risk of nuclear cataract by 13% compared to those assigned to placebo (*P* = .005). There were also 10% fewer cortical cataracts with MVM, although this did not reach statistical significance (*P* = .17). MVMs had no effect on the development of posterior subcapsular cataract (*P* = .85).

It is evident from the studies discussed here that MVM supplements have little effect on the prevention or progression of AMD, with just 1 of 3 studies showing only limited benefit [78]. However, it appears that they can be taken safely in combination with the high-dose antioxidant vitamins that have been shown to reduce the rate of eye disease progression. All 4 of the studies that investigated the effect of MVMs on cataract suggested that MVM supplements have a role in the prevention of cataract, particularly the most common subtype, nuclear cataract.

### Cognition

MVM supplementation has resulted in limited benefits to cognitive performance in some, but not all, studies. A meta-analysis of 10 RCTs (N = 3,200) concluded that daily MVM supplement use by cognitively intact adults significantly improved immediate free recall memory (*P* < .01), with the strongest effect seen for MVM supplements with more constituents, but that MVM supplements had no significant effects on delayed free recall memory or verbal fluency [14]. Two of the studies reviewed suggested possible improvements in mathematical processing ability. One limitation of this analysis is that 7 of the 10 trials studied MVM supplement use for short durations ranging from 6 weeks to 6 months, so the effects of longer MVM use on cognitive function are not known.

In an RCT of healthy adults aged 18 to 86 years, a supplement containing the vitamins folic acid (400 μg), vitamin B_12_ (6 μg), and vitamin E (30 IU alpha-tocopherol), as well as S-adenosylmethionine (400 mg), N-acetyl cysteine (600 mg), and acetyl-L-carnitine (500 mg) improved cognitive performance for the duration of its use only [81]. These latter compounds have a range of physiologic roles in metabolism and are often taken as supplements. Improvements were seen on some parts of the California Verbal Learning Test II (which measures short- and long-term word recall) and the Trail-Making Test (a measure of neuromuscular coordination and executive function) both in the double-blind phase and in an open-label extension. Scores returned to baseline following withdrawal of the supplement, but improved again when the supplement was reintroduced. An increased percentage of participants 74 years of age or older did not show improvement with the supplement product, which may be due to age-related difficulties in adsorption and/or basal nutritional deficiencies, or age-related cognitive decline during the course of the study. The authors concluded that the findings from this particular study support the benefit of nutritional supplements containing vitamins and other biologically active compounds for cognitive performance and that the elderly may require additional supplementation.

An RCT of an Australian supplement containing approximately 50 vitamins, minerals, and herbs found that it improved contextual recognition memory in men aged 50 to 74 years [82]. Contextual recognition memory, a measure of episodic memory, is often the first cognitive function to be impaired in patients with progressive cognitive decline or Alzheimer’s dementia. In a second study, a version of the supplement designed for women improved speed of response on a measure of spatial working memory in women aged 64 to 82 years who had reported memory worsening at screening [83].

The randomized, controlled Mineral and Vitamin Intervention Study, conducted in North-East Scotland, evaluated the cognitive effects of a daily MVM supplement containing 16 vitamins and minerals in 910 persons age ≥65 years [84]. After 1 year, there was no evidence of benefit on tests of immediate memory (digit span forward test) and executive function (verbal fluency test).

Evidence for cognitive benefits of MVMs is limited to a small number of studies showing positive results with only some of the cognitive tests performed. There have been no reports of MVM supplements having a negative impact on cognitive function, and it’s probably reasonable to use MVMs to address nutritional gaps with little, if any, concern for increasing the rate of cognitive decline. However, it should be noted that older persons have likely been exposed to dietary deficiencies for longer periods of time and have nutritional status that is less homogeneous than younger populations, compounded by disease and polypharmacy. As a result, the use of supplements is harder to generalize for populations over 60 years of age.

### Mortality

Concerns have arisen, based on studies employing supplements with individual or combinations of a small number of vitamins, that dietary supplements may possibly be associated with increased mortality. In general, data on the effects of MVM supplements on mortality have been largely reassuring.

In the randomized PHS II study described above, MVM supplementation trended positively, but did not alter total mortality significantly (*P* = .13) [16]. Several large observational studies have similarly shown no increase in risk of mortality among MVM supplement users [72,85,86], as did a meta-analysis of 21 RCTs (RR: 0.98; 95% CI: 0.94–1.02) [15]. Similarly, the Multiethnic Cohort Study of 215,000 ethnically diverse persons living in Hawaii and California who completed a detailed questionnaire on diet, medical history, and lifestyle showed no increase in risk among men or women who used MVM supplements versus nonusers with regard to overall mortality or mortality attributable to CVD, cancer, or all other causes during 11 years of follow-up [85]. The single exception to those findings was in men who used MVM supplements either more than once daily or for <5 years’ duration who had a possible increase in risk of mortality from causes other than CVD or cancer. A study using nationally representative data from NHANES I (N = 10,758) found no evidence that MVM supplements affected mortality [86]. Overall mortality was comparable for regular users compared with nonusers among men (RR: 0.94; 95% CI: 0.82–1.06) and women (RR: 1.02; 95% CI: 0.90–1.17).

One study with contrasting data was the Iowa Women’s Health Study, a nonrandomized, observational trial in postmenopausal women that showed a significant 6% increase in mortality in women taking MVMs [87]. However, the Women’s Health Initiative observational trial, conducted with a similar population of 161,806 postmenopausal women, found no increase in risk of death over 8 years of follow-up among MVM supplement users (HR: 1.02; 95% CI: 0.97–1.07) [62]. Finally, a 5% increase in mortality rate was found in men, but not women, who self-reported use of MVM supplements as part of a large (N = 1,063,023) observational US study [88]; however, death rates were lower among both men and women if they used a combination of MVM supplements and vitamin A, C, or E. Overall, these studies suggest that generally well-nourished populations that take MVMs suffer no significant increased risk of mortality. There are some weaknesses in these analyses of mortality. Firstly, most of these studies rely on self-report of MVM use, so it is not clear if subjects were taking formulations that were age and gender appropriate. Secondly, none of these studies have investigated mortality in a cohort of patients who took an MVM consistently starting at a young age, following them over a lifetime.

### Conclusions and implications for practice

MVMs may help prevent a number of health problems. In bridging nutrient gaps, it is plausible that MVMs help prevent iron-deficiency anemia, neural tube defects, neurological damage in people age 50 years and older, and bone disease by supplementing the diet with iron, folic acid, vitamin B_12_, and vitamin D, respectively.

A recent draft of the US Preventive Services Task Force found that there was no rationale to recommend for or against multivitamin use for primary prevention of CVD or cancer [89]. Although this group noted the significant reductions in cancer incidence in PHS II and SU.VI.MAX, the lack of generalizability to the general population meant evidence was insufficient to recommend routine use of MVM supplementation for primary prevention. The group also noted little consistent evidence to support or refute a role of single and paired vitamins and minerals in the prevention of cancer. A recent review by Comerford suggested that these more recent studies provided moderate evidence suggesting that MVM supplements containing vitamins and minerals at or near the Recommended Dietary Allowance (RDA) are beneficial in reducing the risk of chronic disease in at-risk populations, especially given their demonstrated safety [90].

It is necessary to consider a person’s medical history as well as the quality of their diet when advising on the use of MVMs. For example, for people with a history of cancer, those taking prescription and over-the-counter medications, and those planning surgery should consult their doctors about taking MVMs and other dietary supplements. Likewise, people who take MVMs and other dietary supplements and who regularly consume fortified foods and beverages may consume certain nutrients at levels exceeding the IOM’s UL, which may cause adverse effects.

Even when a diet is well planned, it is not always possible for most people to choose foods containing the recommended amounts of all essential micronutrients, and chronic, relatively minor nutrient shortfalls can cause health problems. The role of MVMs also needs to be considered in the context of macro-nutrient consumption (carbohydrates, protein, and fats) and its effects on digestion, absorption, and bioavailability of vitamins and minerals, as well as the effect of the balance of macro-nutrient intake on metabolism and the need for different vitamins and minerals. While MVM supplements cannot replace eating adequate amounts of a variety of foods, they may be particularly beneficial to people who have poor nutrition for a variety of reasons, including inadequate intake of foods from all the food groups, advanced age, and chronic illness.

When choosing an MVM product, consumers should take a preparation that is tailored to their age, gender, family history/risk factors, and stage of life, including the childbearing years and the senior years. Wherever possible, the specific MVM formulation selection should be based on a nutritional assessment that identifies deficiencies as well as inadequate or erratic intake that can put a person at risk for deficiencies. The decision to take an MVM, like the decision to take any drug or supplement, should consider whether any potential risks outweigh the benefits. For example, smokers and, possibly, former smokers should avoid MVMs with large amounts of β-carotene or vitamin A, because studies have linked these nutrients to an increased risk of lung cancer in this population [42,43]. Taking excess vitamin A (as preformed retinol but not β-carotene) during pregnancy can increase the risk of birth defects, so women capable of conceiving should consider the advice to limit daily consumption of preformed vitamin A to 10,000 IU/d (3,000 μg/d) or less [91]. Likewise, the Centers for Disease Control and Prevention recommends all women capable of becoming pregnant take 400 μg folic acid daily to help prevent birth defects [4]. Most healthy adult males and postmenopausal women should also avoid MVMs with more than 8 mg of iron and limit their total iron intake to 45 mg/d [91].

When deciding whether to recommend the use of dietary supplements, it is important to consider the benefit:risk ratio. Current data suggest minimal, if any, risk associated with MVM preparations containing 10 or more vitamins and minerals at recommended daily intake levels in healthy people and a possibility of modest benefits that include a reduced risk of cancer and nuclear cataract, for a relatively low financial cost.

## Competing interests

Elizabeth Ward received an honorarium from Pfizer in connection with the development of this manuscript.

## Authors’ contributions

The author alone was responsible for the content and writing of the manuscript.
